# Supplementary material for: Advancing Cancer Therapy Predictions with Patient-Derived Organoid Models of Metastatic Breast Cancer
Source: Cancers (Basel). 2023 Jul 13;15(14):3602. doi: 10.3390/cancers15143602 (PMC10377262; doi:10.3390/cancers15143602)
Supplement: Supplementary file 1 [file cancers-15-03602-s001.zip › cancers-2488665-supplementary.pdf]

cancers

MDPI

Supplementary Materials: Advancing Cancer Therapy Predictions with Patient-Derived Organoid Models of Metastatic Breast Cancer

Cansu E. Önder, Teresa J. Ziegler, Ronja Becker, Sara Y. Brucker, Andreas D. Hartkopf, Tobias Engler, André Koch

Table S1. List of BC patient data from whom the organoid lines were established. All patients were female aged between 52 and 79. Abbreviations: ILC (invasive lobular carcinoma), NST (no special type).

MBC-PDO	#02	#03	#04	#05	#06	#07
**Primary tumor**	ILC (1993)	NST (2008)	NST (2009)	ILC (2013)	NST (2019)	NST (2019)
**Therapy of primary tumor**	No adjuvant drug therapy	Trastuzumab; Aromasin; Carboplatin; Taxol; Avastin (until 2019)	FEC + Docetaxel; Tamoxifen (until 2013)	Not known	Epirubicin/Cyclo-phosphamide (aborted)	Doxorubicin
**Metastasis**	Uterus and perito-neal metastasis (2010)	Hepatic and pleural metastasis (2019)	Lymph nodes (2017) Hepatic metasta-sis (2021)	Peritoneal metastasis (2021) Osseus, ovarian and hepatic me-tastasis (2021)	Hepatic and os-seus metastasis (2020) Pulmonary and medullary metas-tasis (2021)	Cutaneous, lym-phogenic and os-seus metastasis (2020-2021)
**Therapy of Metastasis**	Letrozole; Carboplatin + Taxol; Letrozole (until 2019)	Avastin	Ribociclib + Letrozole; Palbociclib + Letrozole	See "follow-up therapy"	Letrozole + Goserelin + Abemaciclib	Paclitaxel; Eribu-lin; Abemaciclib + Letrozole
**Therapy prior to drainage**	Palbociclib + Anastrozole (2019)	Avastin; Nab-Paclitaxel; Eribulin (2020)	Abemaciclib + Fulvestrant (2020-2021)	See "follow-up therapy"	Paclitaxel (2021)	Capecitabin (2021)
**Drainage**	Ascites (2020)	Pleura (2021)	Pleura (2021)	Ascites (2021)	Pleura (2021)	Pleura (2021)
**Follow-up therapy**	None, patient did not survive	Palbociclib + Letrozole; Vinorelbine + Trastuzumab + Pertuzumab	Everolimus + Aromasin; Doxorubicin	Nab-Paclitaxel + Trastuzumab + Pertuzumab; Fulvestrant + Trastuzumab + Pertuzumab; Abemaciclib + Trastuzumab + Fulvestrant	None, patient did not survive	Carboplatin + Olaparib; Olaparib
**Additional information**	-	Hepatic and pleural metastasis (2019): NST infiltration, *PIK3CA* H1047R mutation	-	Mamma Biopsy (2021): *PIK3CA* E545K mutation	-	NST (2019): *BRCA1/2* deletion, *AKT1* E17K mutation

Table S2. Composition of Breast Cancer Medium (BCM). Composition was previously described [24, 27].

Component	Final concentration	Company	Catalog number
L-WRN conditioned medium	50%	Home-made	-
Neuregulin 1	5 nM	Peprotech (Cranbury, NJ, USA)	100-03
FGF7	5 ng/ml	Peprotech (Cranbury, NJ, USA)	100-19
FGF10	20 ng/ml	Peprotech (Cranbury, NJ, USA)	100-26
EGF	5 ng/ml	Peprotech (Cranbury, NJ, USA)	AF-100-15
A83-01	500 nM	Tocris Bioscience (Bristol, UK)	2939
Y-27632	5 µM	Hölzel Diagnostika Handels GmbH (Köln, Germany)	TMO-T1725-50 mg
SB202190	500 nM	Sigma-Aldrich (St. Louis, MO, USA)	S7067
B-27™ supplement	1x	Thermo Fisher Scientific (Waltham, MA, USA)	17504-44
Nicotinamide (NIC)	5 mM	Sigma-Aldrich (St. Louis, MO, USA)	NO636
N-Acetylcysteine (NAC)	1.25 mM	Sigma-Aldrich (St. Louis, MO, USA)	A9165-5G
Primocin	50 µg/ml	InvivoGen (San Diego, CA, USA)	Ant-pm-1
AdvDMEM +++ (1% Pen/Strep, 1x GlutaMAX™-I, 10 mM HEPES)	1x	Thermo Fisher Scientific (Waltham, MA, USA)	12634-028; 15140-122; 35050-038; 15630-056

Table S3. List of inhibitors applied in 3D drug screenings.

Inhibitor	Company	Catalog Number
Abemaciclib	Hycultec	HY-16297A
Afatinib	Hycultec	HY-10261
Alpelisib	Hycultec	HY-15244
Capivasertib	Selleckchem	S8019
Everolimus	Selleckchem	S1120
Gemcitabine	Selleckchem	S1714
Ipatasertib	Hycultec	HY-15186
Lapatinib	Hycultec	HY-50898
Neratinib	Hycultec	HY-32721
Olaparib	Hycultec	HY-10162
Paclitaxel	Sigma	T7191
Palbociclib	Hycultec	HY-50767
Pictilisib	Selleckchem	S1065
Tucatinib	Hycultec	HY-16069

Table S4. List of antibodies used for IHC stainings.

Antibody target	Dilution	Company	Catalog number
CDH1 (clone NCH-38)	1:50	Agilent Technologies	M3612
EpCAM (clone Ber-EP4)	1:50	Agilent Technologies	M0804
ERα (F-10)	1:50	Santa Cruz Biotechnology	SC-8002
GATA3 (HG3-31)	1:200	Santa Cruz Biotechnology	SC-268
HER2 (c-erbB-2)	1:500	Agilent Technologies	A0485
Ki67	1:500	Abcam	AB16667
p53 Protein (DO-7)	1:50	Agilent Technologies	M700129-2
phospho-AKT (Ser473)	1:200	Cell Signaling	4060S
PR (Clone PgR 6369	1:50	Agilent Technologies	M356929-2

Table S5. Primers used for the amplification of hotspot mutations and for sequencing. All primers were purchased from Sigma-Aldrich. *Reverse primer for *PIK3CA* E542 and E545 mutation was designed based on a previous publication, to eliminate the amplification of a pseudogene [29].

Primer	Sequence (5′ → 3′)	Application
Forward primer for *PIK3CA* mutation (exon 9 covering E542 and E545)	AATCTGGTCTTGTTGTTGGCT	PCR
Reverse primer for *PIK3CA* mutation * (exon 9 covering E542 and E545)	CCATTTTAGCACTTACCTGTGAC	PCR
Sequencing primer for *PIK3CA* mutation (exon 9 covering E542 and E545)	ATCATCTGTGAATCCAGAGGGGA	Sequencing
Forward primer for *PIK3CA* mutation (exon 20 covering H1047)	TGGTAAGAGAAGTGAGAGAGGA	PCR
Reverse primer for *PIK3CA* mutation (exon 20 covering H1047)	CAGCCTTTGTTGTGTCCACATT	PCR
Sequencing primer for *PIK3CA* mutation (exon 20 covering H1047)	CGACAGCATGCCAATCTCTTC	Sequencing
Forward primer for *AKT* mutation (exon 1 covering E17)	CTGGTTGATTGGGGAATGCT	PCR
Reverse primer for *AKT* mutation (exon 1 covering E17)	AAATCTGAATCCCGAGAGGC	PCR
Sequencing primer for *AKT* mutation (exon 1 covering E17)	TCGCTGGCCCTAAGAAACAG	Sequencing

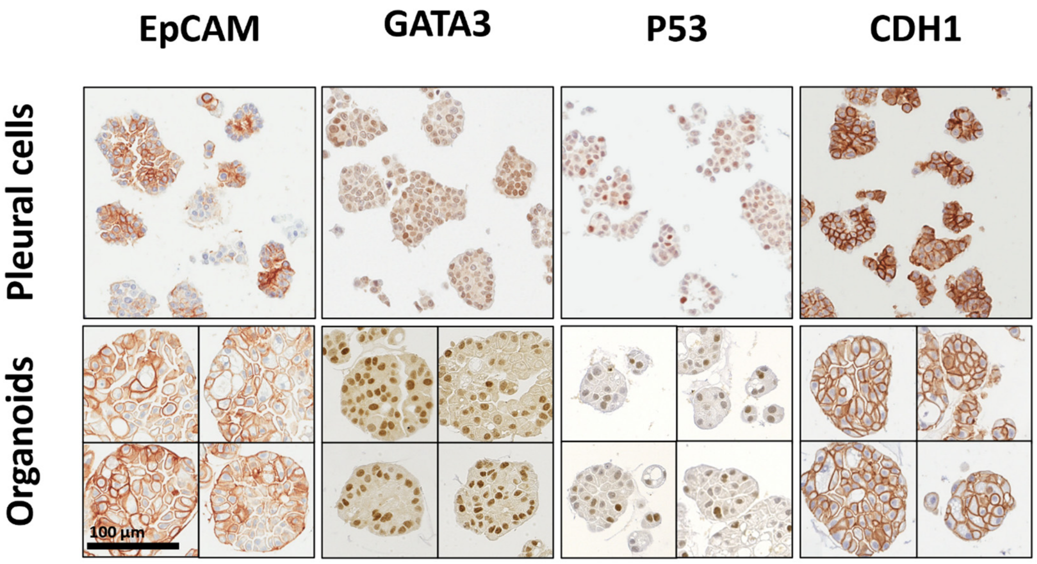

Figure S1. Histological characterization of pleural BC cells and organoids derived from pleural effusion. IHC staining of MBC-PDO #03 with antibodies against EpCAM, GATA3, P53, and CDH1. The expression pattern of pleural cells is retained in organoids (P3). Scale bar: 100 µm.

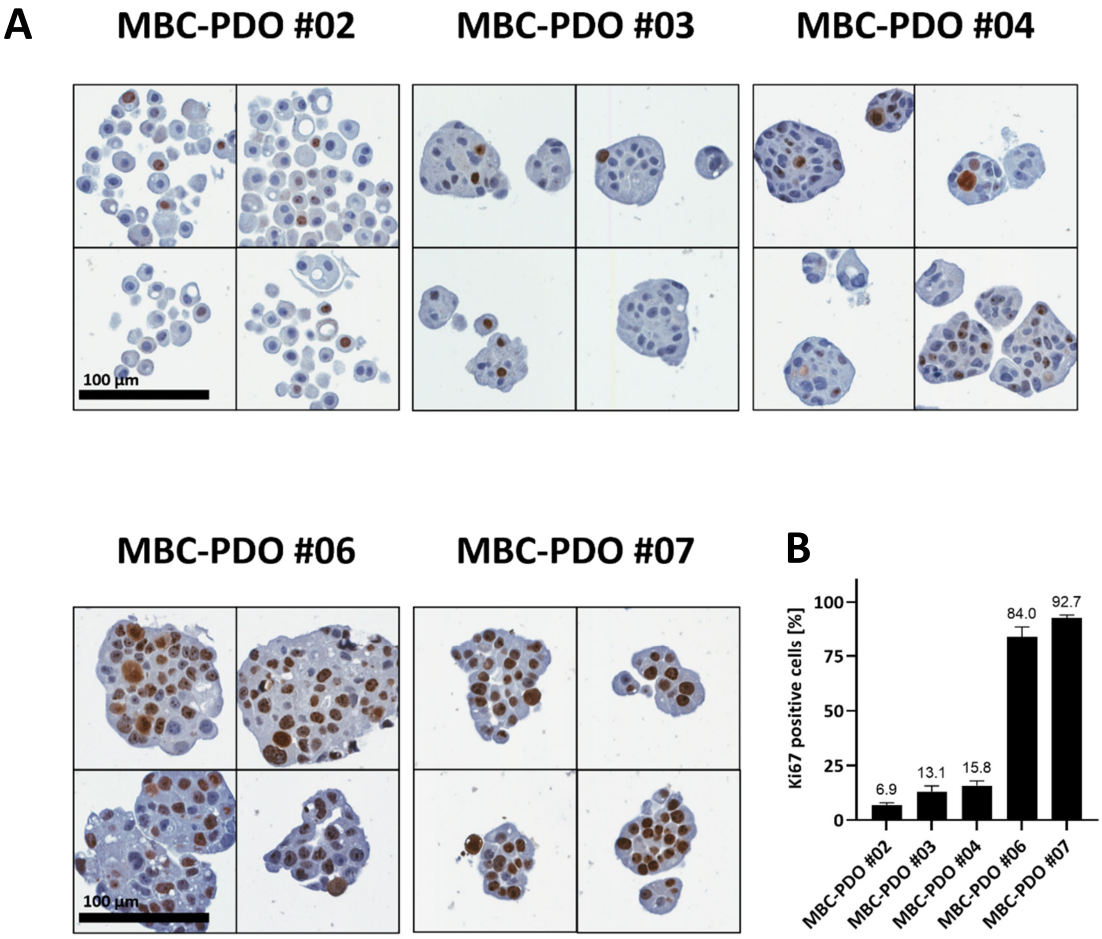

Figure S2. IHC staining of the proliferation marker Ki67 in organoids. **(A)** IHC stainings of FFPE samples from organoids. Brown nuclei indicate positivity. Scale bar: 100 µm. **(B)** Quantification of Ki67 positive cells. Ki67-stainings were quantified by counting 150 cells of each organoid line and determining the ratio of Ki67-positive cells. Quantification was done in triplicates.

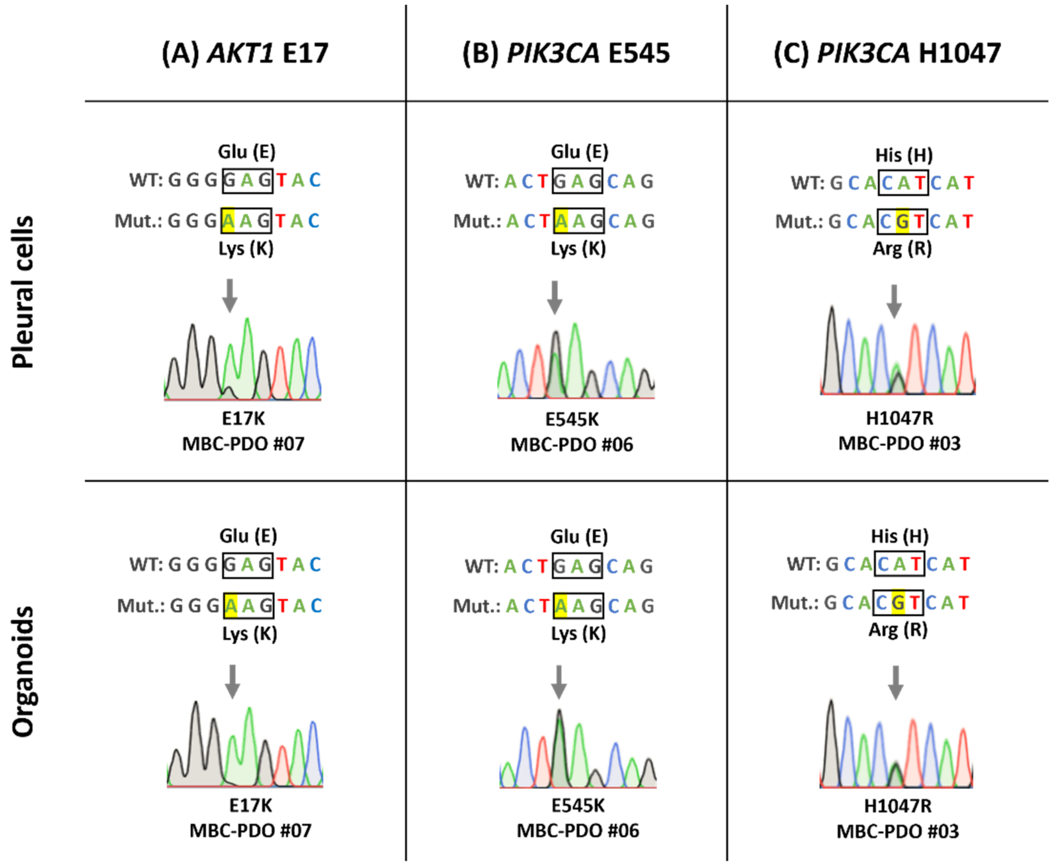

Figure S3. Examples of mutations in *AKT1* and *PIK3CA*. Mutation analysis of three common hotspots in *AKT1* and *PIK3CA* genes. PCR-amplified DNA samples were sequenced by Sanger sequencing and depicted in chromatograms. Shown are wild type (WT) sequences and the sequence of mutated samples. DNA bases are represented by the following colors: guanine (G) in black, cytosine (C) in blue, adenine (A) in green, thymine (T) in red. **(A)** Homozygous mutation of *AKT1* E17K in MBC-PDO #07 pleural cells and organoids. **(B)** Heterozygous mutation of *PIK3CA* E545K in MBC-PDO #06 pleural cells and organoids. **(C)** Heterozygous mutation of *PIK3CA* H1047R in MBC-PDO #03 pleural cells and organoids.

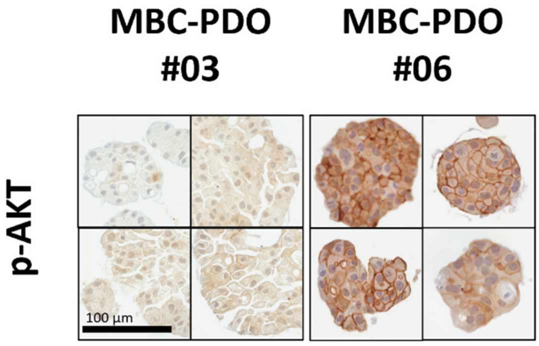

Figure S4. IHC-staining of p-AKT. MBC-PDO #03 and #06 organoids were stained with an antibody against p-AKT to demonstrate the activity of the PI3K-AKT signaling pathway. Cytoplasmic expression of p-AKT was observed in MBC-PDO #06. Scale bar: 100 µm.
